# Unlocking Clinical Insights and the Life-Changing Impact of Low-Vision Devices in Primary Open-Angle Glaucoma Patients

**DOI:** 10.7759/cureus.70458

**Published:** 2024-09-29

**Authors:** Gaurav Paramasivan, Sarika Gopalakrishnan, Mona Khurana, Rajiv Raman

**Affiliations:** 1 Department of Optometry, Sankara Nethralaya, Chennai, IND; 2 Department of Low Vision Care, Sankara Nethralaya, Chennai, IND; 3 Department of Smt. Jadhavbai Nathmal Singhvee Glaucoma Services, Sankara Nethralaya, Chennai, IND; 4 Department of Vitreoretinal Services, Sankara Nethralaya, Chennai, IND

**Keywords:** low vision care clinic, low vision devices, primary open angle glaucoma, visual field, visual impairment, visual rehabilitation

## Abstract

Objective

To elucidate the clinical profile of visual impairment (VI) and the effect of low-vision devices (LVD) among patients with primary open-angle glaucoma (POAG).

Methodology

A retrospective observational study was used. Case records of patients who reported to the low-vision care clinic with POAG were recruited for the study. Glaucoma was defined according to the International Society for Geographical and EO classification. Demographic data, visual acuity (LogMAR), clinical diagnosis, visual fields, occupation, and LVD prescribed were extracted from the records. The visual acuity pre- and post-use of LVD was compared. Visual acuity improvement while using the LVD was noted.

Results

Of the 200 POAG cases, 20% (n=40) had severe, 15.5% (n=31) had profound (<3/60), 20.5% (n=41) profound (<1/60), and 26.5% (n=53) had total blindness in the VI category. Significant improvement was noted for both distance and near vision (p=0.004 and p=0.000, respectively) for single-vision glasses, and significant improvement (p=0.000) was also noted for patients corrected for both distance and near correction (bifocal/progressive). Patients with severe visual field loss (p=0.004) and with a visual field defect of <10 degrees (p=0.000) both showed a significant improvement in near vision with low-vision rehabilitation. The proportion of patients earning showed improvement following LVD use compared to those who did not have self-earning (60% vs. 40.24%, p=0.0116).

Conclusion

LVD significantly improved near vision in POAG patients with advanced glaucoma, a visual field defect of less than 10 degrees. To effectively manage POAG patients with low vision, it is vital to understand both the ocular diseases causing VI and the patient's visual requirements and provide timely management.

## Introduction

Globally, approximately 57.5 million individuals are affected by primary open-angle glaucoma (POAG), with a prevalence rate of 2.2% [1]. Untreated glaucoma can lead to irreversible optic nerve head damage resulting in visual field defects, which over time can progress to irreversible blindness [2]. Since glaucoma is a silent disease, patients often do not seek timely treatment and are disabled by varying degrees of permanent visual impairment (VI) [3].

Patients with VI due to glaucoma may have the following symptoms: a requirement for greater illumination than usual, blurry vision, diminished contrast sensitivity, glare, difficulty seeing objects on one or both sides and an inability to perceive boundaries and differentiate between colors [4]. An increased risk of falls and problems in reading, driving, and mobility have also been reported. They are managed and rehabilitated using various low-vision devices (LVD) and techniques. Low-vision care (LVC) - the stanchions of care in patients with irreversible visual loss due to glaucoma are integral to visual rehabilitation. Magnification devices are one of the most commonly employed means of visual rehabilitation for glaucoma [5-7]. Most of the treatment approaches are founded on one of the following four keystones: increasing the relative size of the object itself, increasing its relative size by reducing its relative distance, angular magnification (telescopes), and amplification by electronic projection [8].

Patients with glaucoma have been observed to have benefited from LVD, which in turn enhanced their quality of life. Previous studies have demonstrated the benefit of LVD in patients with VI, including those with glaucoma [6,9,10]. Monira et al. showed the preference patterns of LVD in patients with glaucoma and identified factors affecting the choice of LVD. However, the improvement in the quality of vision after using LVD in patients with POAG has not been reported [11].

The present study aims to analyze the clinical profile of VI in patients with POAG and to further assess the effect of LVD among patients with POAG.

## Materials and methods

This retrospective observational study was conducted in the LVC Clinic in Sankara Nethralaya, Chennai, India. In the clinic, an LVC specialist and optometrists trained in low-vision management offer services to help patients learn how to use their vision to the fullest potential in their clinic by assessing their visual acuity, contrast, field of vision, eye-hand coordination, and color vision and providing them with devices, rehabilitation, and a disability certificate letter after the comprehensive evaluation and treatment. A retrospective review of case records of patients who were referred to the LVC clinic in a year at a tertiary eye care institute in India was conducted. We included all patients diagnosed with POAG who were referred to the LVC clinic during the study period. A total of 5327 records were reviewed, of which 200 patients were identified as suitable for inclusion. Glaucoma was defined according to the International Society for Geographical and EO classification [12]. Patients with retinal pathologies like diabetic retinopathy, age-related macular degeneration, total cataracts, vascular occlusions, and other subtypes of glaucoma were excluded. Institutional review board approval was obtained to analyze the hospital-based data, and the tenets of Helsinki were followed.

Data collection

The low-vision assessment was conducted at a dedicated LVC. The following data was collected: demographics, clinical features, presenting distant (by Bailey Lovie chart) and near visual acuity (Minnesota low-vision reading chart), details of the LVD prescribed (optical and non-optical), visual fields and the distant, and near visual acuity with the LVD. Different language charts, including Tamil, English, Hindi, and Telugu, were used based on the patient’s preference, educational level, and comfort. The tumbling E chart was used for illiterate patients based on their preferences. Visual fields were performed using the Humphrey visual field analyzer 3 (HFA, Carl Zeiss Meditec Inc., Dublin, USA). POAG patients were classified as mild, moderate, and severe based on the Hodapp-Parrish-Anderson classification [13].

The pre- and post-LVD vision was also compared in subgroups based on the extent of the visual field and the severity of glaucomatous field defect.

Types of LVD utilized

Distance optical devices were used to magnify objects at a distance of three meters or more, whereas near optical devices were used to magnify printed material and proximate objects. Different types of optical devices were used, either in isolation or in combination, to improve the visual acuity of patients with low vision.

The patients were given a trial of a single or a combination of low-vision optical and non-optical devices, depending on their presenting visual acuity, and the maximum improvement in the visual acuity was noted. A detailed demonstration and explanation regarding the use of the device and adaptation training with the preferred device were given to the patients to help achieve independent device handling. When required, multiple devices (optical and non-optical) were also prescribed for better visual improvement. In addition to the LVD prescription, the instruction manual of the prescribed device was also provided for assistance on the use of the device.

Levels of visual impairment

Low vision was defined based on recommendations by the WHO using visual acuity of the better eye with the best possible correction as the parameter of assessment [14-16].

Statistical analysis

Data was entered, coding was created, and data cleaning was done in Microsoft Excel 2010. Statistical analysis was performed using IBM SPSS Statistics for Windows, Version 20 (Released 2011; IBM Corp., Armonk, New York, United States). The normality of the data was tested using the Shapiro-Wilk test. To compare two paired groups in non-normally distributed data, the Wilcoxon signed-rank test was used. The alpha value was set as <0.05, considered statistically significant.

## Results

Out of 200 patients, 53 patients had no perception of light; hence, those patients were excluded from the analysis. These patients underwent non-optical interventions based on their requirement task. The median age of the patients was 56 (36 IQR) years. Amongst them, 10% (n=20) were less than 20 years of age, 19.5% (n=39) were between 21 and 40 years of age, 29% (n=58) were between 41 and 60 years of age, 37% (n=74) were between 61 and 80 years of age and 4.5% (n=9) belonged to more than 80 years of age. The majority of patients (77% (n=154)) were male. Most (57% (n=114)) belonged to the rural population, and 20.5% (n=41) were retired in their occupation (Table 1).

**Table 1:** Demographic details of patients IQR: interquartile range; n: number; PL: perception of light Occupation was categorized based on the patient’s age and occupation.

Categories	Subcategories	Values n (%)
Age	Median (IQR)	56 (36)
Gender	Male	154 (77)
	Female	46 (23)
Geographical status	Rural	114 (57)
	Urban	86 (43)
Occupation	Student	32 (16)
	Discontinued studies	7 (3.5)
	Unemployed	12 (6)
	Employed	55 (27.5)
	Housewife	18 (9)
	Retired	41 (20.5)
	Self-business	35 (17.5)
Source of income	Financially-independent	131 (65.5)
	Financially-dependent	69 (34.5)
MD on visual field examination (decibels)	Median (IQR)	-21.63 (17.15)

Severe to no perception of light in the VI category was present in 82.5% (n=165) of patients (Table 2). Advanced glaucoma was present in 50.5% (n=101) patients.

**Table 2:** Severity of visual impairment

Category	Best corrected visual acuity of the better eye	Number (percentage) (n=200)
Category 0	Mild VI with visual acuity better than 6/18	9 (4.5)
Category 1	Moderate VI with worse than 6/18-6/60	26 (13)
Category 2	Severe VI with worse than 6/60-3/60	40 (20)
Category 3	Profound VI with worse than 3/60	31 (15.5)
Category 4	Profound VI with worse than 1/60	41 (20.5)
Category 5	Blindness with no perception of light	53 (26.5)
VI: visual impairment; n: number		

**Figure 1:**
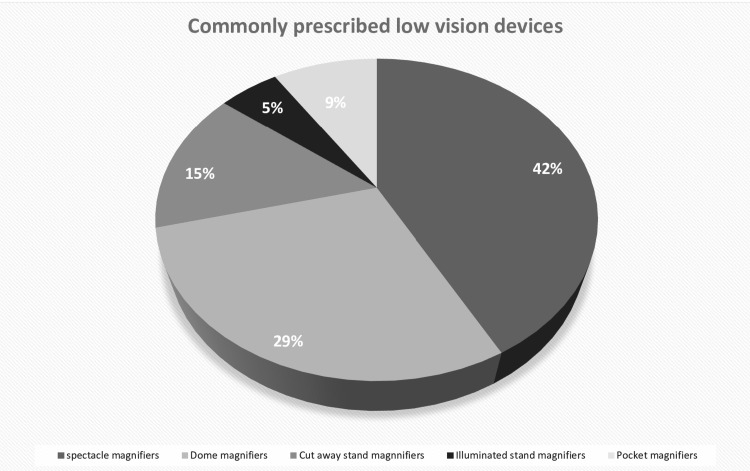
Commonly prescribed low-vision devices among primary open-angle glaucoma patients

Spectacle magnifiers (49% (n=32)) and dome magnifiers (29% (n=22)) were the most prescribed devices among the patients (Figure 1).

Though spectacle magnifiers and dome magnifiers were prescribed most commonly, only spectacle magnifiers showed significant near vision improvement of 0.7 (0.4 IQR) to 0.5 (0.5 IQR) visual acuity; p=0.006 and there is no significant difference in visual acuity noted for dome magnifier, 0.4 (0.2 IQR) to 0.3 (0.1 IQR) visual acuity; p=0.054, respectively.

For 53 patients with no perception of light, non-optical interventions were done. Nine patients were guided for educational support and five patients were guided for vocational training at the National Association for the Blind. Three patients were advised for environmental modification/contrast enhancement, three were advised to be seated in the front row constantly, and one patient was advised to use a thick pen while writing. All these patients were trained to identify currency and notes by touching them with the help of NOTEX.

Optical interventions were recommended for 147 patients. Of these, 44.21% (n=65) had improvement in vision with LVD, 14% (n=20) had improvement in distance vision, and 49.30% (n=71) in both distance and near vision following the prescription of glasses to correct their refractive error. Out of 147 patients, 21 patients (14.28%) were prescribed assistive devices (non-optical devices) and two patients (14%) were prescribed non-optical devices (clip-on filter).

**Figure 2:**
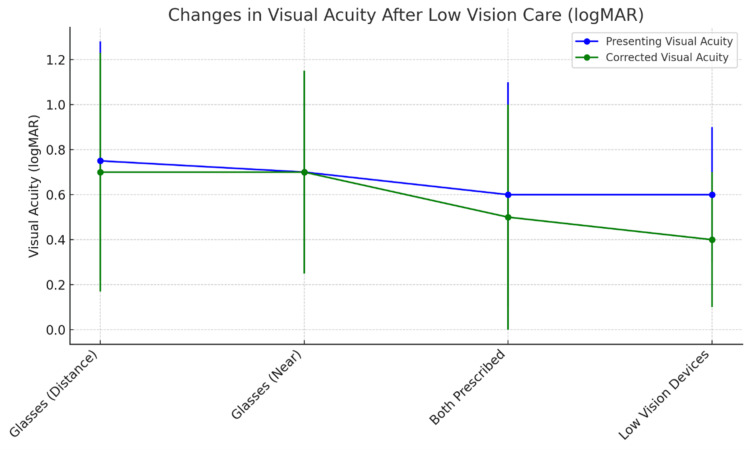
Changes in visual acuity in eyes with primary open-angle glaucoma following low-vision care

Significant improvement in the median near vision was noted (Table 3) using LVD (p=0.002). A pictorial representation was also provided along with Figure 2. Significant improvement in the median distance vision was also noted with the use of glasses (p=0.011). However, there was no change in the median near vision with glasses (p=0.180).

**Table 3:** Changes in visual acuity in eyes with primary open-angle glaucoma following low-vision care IQR: interquartile range p-values presented in the table were calculated using the Wilcoxon signed-rank test. Negative values indicate an improvement in visual acuity (a reduction in logMAR value).

Methods of correction	Categories	Presenting visual acuity before low-vision care (median (IQR))	Best corrected visual acuity after low-vision care (median (IQR))	p-value	Change (median (IQR))
Glasses	Distance visual acuity (logMAR)	0.75 (0.53)	0.70 (0.67)	0.011	-0.05 (0.14)
	Near visual acuity (logMAR)	0.70 (0.45)	0.70 (0.40)	0.180	0.00 (0.05)
Both prescribed		0.60 (0.5)	0.5 (0.3)	0.000	-0.10 (0.20)
Low-vision devices	Near visual acuity (logMAR)	0.6 (0.3)	0.40 (0.4)	0.000	-0.20 (0.10)

**Figure 3:**
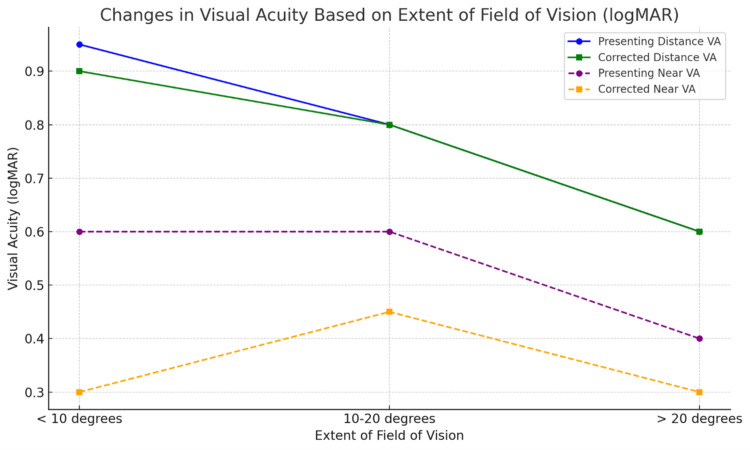
Change in visual acuity following low-vision care (low-vision devices) according to severity and extent of residual field of vision of primary open-angle glaucoma patients

In our study, in the classification of patients based on visual field defects (<10 degrees, 10-20 degrees, and >20 degrees), 75% of patients (n=150) had advanced glaucomatous visual field defects. With the prescription of LVD, significant improvement was seen in near vision in patients with a visual field of less than 10 degrees, and in those with a visual field between 10 and 20 degrees (Table 4). A pictorial representation was also provided along with Figure 3. However, there was no significant improvement in the near vision in patients (n=6) with a visual field of >20 degrees (p=0.577). No significant improvement in vision was noted in distance vision.

**Table 4:** Change in visual acuity following low-vision care (low-vision devices) according to severity and extent of residual field of vision of primary open-angle glaucoma patients IQR: interquartile range p-values presented in the table were calculated using the Wilcoxon signed-rank test. 0 indicates patients who did not undergo field examination; negative values indicate an improvement in visual acuity (a reduction in logMAR value).

Category	Distance visual acuity					Near visual acuity			
		Presenting visual acuity before low-vision care (logMAR)	Best-corrected visual acuity after low-vision care (logMAR)	p-value	Change (logMAR)	Presenting visual acuity before low-vision care (logMAR)	Best-corrected visual acuity after low-vision care (logMAR)	p-value	Change (logMAR)
Extent of field of vision	<10 (n=59)	0.95 (0.5)	0.90 (0.42)	0.954	-0.05	0.60 (0.3)	0.30 (0.25)	0	-0.3
	10-20 (n=15)	0.80 (0.425)	0.80 (0.502)	0.492	0	0.60 (0.325)	0.45 (0.4)	0.024	-0.15
	>20 (n=6)	0.60 (0.1)	0.60 (0.4)	0.109	0	0.40 (0.2)	0.30 (0.2)	0.577	-0.1
Severity of glaucoma	Mild (n=10)	0.55 (0.1)	0.60 (0.475)	0.109	0.05	0.55 (0.35)	0.30 (0.375)	0.101	-0.25
	Moderate (n=7)	0.80 (0.585)	0.80 (0.57)	0.713	0	0.40 (0.35)	0.30 (0.1)	0.109	-0.1
	Severe (n=42)	1.00 (0.68)	1.00 (0.7)	0.968	0	0.50 (0.3)	0.40 (0.4)	0.004	-0.1

**Figure 4:**
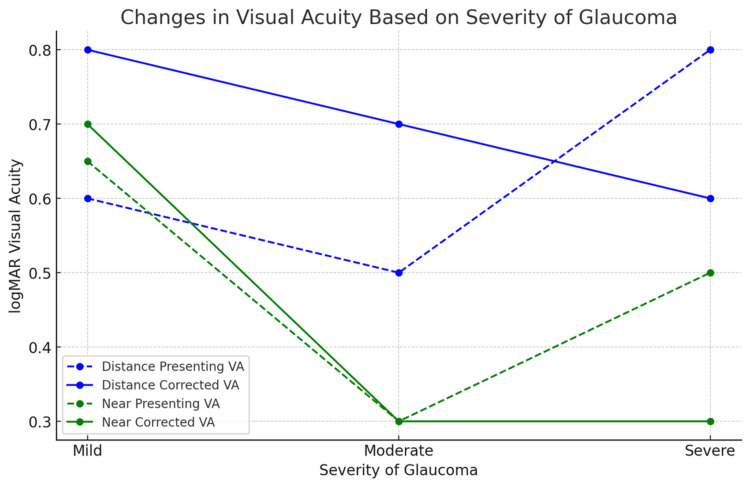
Change in visual acuity following low-vision care (glass prescription) according to severity and extent of residual field of vision of primary open-angle glaucoma patients

Given the severity of glaucoma, visual fields could not be performed in 23 patients with advanced glaucoma due to poor visual acuity. Patients (n=42) with advanced glaucoma (average MD -21.63±16.77 decibels) showed a significant improvement in near vision (p=0.004) with the use of LVD (Table 5). A pictorial representation was also provided along with Figure 4. However, no significant improvement in vision was observed for distant vision. No significant improvement in vision was noted in patients with both mild and moderate glaucoma.

**Table 5:** Change in visual acuity following low-vision care (glass prescription) according to severity and extent of residual field of vision of primary open-angle glaucoma patients IQR: interquartile range p-values presented in the table were calculated using the Wilcoxon signed-rank test. 0 indicates patients who did not undergo field examination; negative values indicate an improvement in visual acuity (a reduction in logMAR value).

Category	Distance visual acuity				Near visual acuity			
	Presenting visual acuity before low-vision care (logMAR)	Best-corrected visual acuity after low-vision care (logMAR)	p-value	Change (logMAR)	Presenting visual acuity before low-vision care (logMAR)	Best-corrected visual acuity after low-vision care (logMAR)	p-value	Change (logMAR)
Extent of field of vision								
<10 (n=70)	0.90 (0.45)	0.85 (0.46)	0.012	-0.05	0.60 (0.3)	0.30 (0.0)	0	-0.3
10-20 (n=22)	0.65 (0.65)	0.55 (0.55)	0.222	-0.1	0.45 (0.3)	0.30 (0.275)	0.187	-0.15
>20 (n=8)	0.60 (0.2)	0.60 (0.3)	0.197	0	0.45 (0.3)	0.35 (0.35)	1	-0.1
Severity of glaucoma								
Mild (n=10)	0.60 (1.195)	0.80 (0.955)	0.293	0.2	0.65 (0.475)	0.70 (0.475)	0.216	0.05
Moderate (n=5)	0.50 (0.5)	0.70 (0.5)	0.655	0.2	0.30 (0.0)	0.30 (0.0)	1	0
Severe (n=42)	0.80 (0.425)	0.60 (0.525)	0.054	-0.2	0.50 (0.35)	0.30 (0.1)	0.003	-0.2

Significant improvement in visual acuity; p=0.012 for distance and near vision (p<0.001) was noted in eyes with visual field <10 degrees. No significant improvement was noted for distance visual acuity; p=0.054, but near vision showed significance as 0.50 (0.35 IQR) to 0.30 (0.1 IQR) visual acuity; p=0.003 in advanced POAG.

Out of 147 patients, 21 patients (14.28%) were prescribed with assistive devices (non-optical devices). Among them, 15 (71.42%) patients with Niki CCTV, four (19.04%) patients with Ruby CCTV, one (5.0%) patient with Senorita CCTV, and one patient with Snow 7 HD (5.0%) as assistive devices were prescribed. Among this one (5.0%) patient had mild VI, four (19.04%) patients had moderate VI, three (14.28%) patients had severe VI, three (14.28%) patients had profound VI (<3/60), and 10 (48%) patients had profound VI (<1/60). Sixteen (76.19%) patients had a visual field of less than 10 degrees and six (29%) patients had a visual field between 10 and 20 degrees in the better eye.

Ninety (45%) patients had difficulty with mobility; the head scanning technique was taught to those patients. About 28 (14%) patients had photophobia and those patients were prescribed tinted glasses. Seventy-one (19%) patients aged 12 years to 90 years of age had trouble with computer usage. Those patients were taught computer modification techniques like contrast enhancement, bigger font size, bigger mouse pointer, magnification, and talkback options.

Financially independent patients benefited more from LVD as compared to those who were dependent on others (65.5% (n=131)) versus (34.5% (n=69); p=0.000). Male patients benefited more from LVD as compared to female patients (77% (n=154)) versus (23% (n=46); p=0.000). Rural patients benefited more from LVD as compared to urban patients (57% (n=114)) versus (43% (n=86); p=0.005). There was no other significant difference in demographics among patients who benefit from LVD compared to those who did not show improvement. All the patients who showed improvement with LVD and glasses purchased the prescribed devices and glasses.

## Discussion

We analyzed the clinical profile of VI in patients with POAG and assessed the effect of LVD among patients with POAG. We found that the spectacle magnifier and dome magnifier were the most preferred LVD in patients with POAG.

Our study shows that 82.5% (n=165) of patients with POAG referred to the low-vision clinic had severe to total blindness in the VI category. The majority 75% (n=150), had an advanced glaucomatous visual field defect and a visual field of less than 10 degrees. The male preponderance of 77% (n=154) was seen in the gender-wise distribution of POAG patients. This may be because the accessibility to healthcare was higher among males when compared to females. The majority of our patients were from a rural area. Pabon et al. reported that, on average, 23 patients with glaucoma attended the low-vision clinic over about 13 years [17]. More than half were more than 80 years of age, with a median age of 82 years. Only 13% of the total 274 patients had a best corrected visual acuity of <20/200. However, the median age of your study population was younger; nearly 82% of the total 200 patients had a best corrected visual acuity of <20/200, and the majority had moderate to advanced glaucoma. In terms of vision, a significant improvement in near vision was noted with the use of LVD.

There has been an increase in the prevalence of POAG per decade of age for South Asians [18]. Approximately 70% of the world's cases of POAG have taken nascence in developing countries - a fact that augments the significance of the outcomes of this study [19].

Some studies have shown a higher prevalence of primary open-angle glaucoma in men, whereas others have found no gender difference [20-22]. Ideally, our study showed male preponderance only. In terms of VI changes, it seems almost incontrovertible that LVD help in the rehabilitation of patients with advanced visual field defects and severe VI vision given that most of the patients (46%) benefited from LVD and 51.5% from glasses in our study, which can be attributed to the relatively higher number of glaucoma patients.

Monira et al. [11] highlighted in their study that of 67 glaucoma patients, 32.5% of the patients were prescribed a spectacle magnifier as an LVD followed by a handheld magnifier, a finding that our study has found favorably. In our study, the same preference pattern was noted for the spectacle magnifier.

In addition to optical devices, our 66 patients (33%) were also advised the use of additional illumination during reading, suggestions to improve contrast in their environment, and the use of NOTEX for their daily routine.

Monira et al. recommended that to find the preference pattern of LVD, the severity of visual disability is mandatory [11]. In our study, changes in vision in subgroups based on both the extent of the visual field and the severity of glaucomatous damage were analyzed. The majority of the cases referred to the LVC had advanced glaucoma. Patodia et al. also reported an increase in the overall visual ability in the 10 glaucoma patients (primary and secondary open-angle glaucoma) in the LVD arm of their pilot randomized control trial as compared to the control arm (total of 16 patients) [8]. However, they did not report the change in visual acuity.

We found a significant improvement in vision in patients with POAG (p<0.001) in near vision with the use of LVD. This shows that patients with advanced visual field defects benefited well from the use of the LVD prescribed. That would, without doubt, fuel their satisfaction with life to the higher echelons and turn them into individuals with far greater productivity than they would be. In our study, more patients who were financially independent showed improvement following LVD as compared to those dependent on others. This gives an impact that the economic status (source of income) plays a role in the usage of required LVD usage and thereby visual prognosis. Patients with this profile can be referred to LVC clinics, as they are more likely to benefit.

The greatest fortes of this study are the standard, methodical procedures that were put in place at the LVC clinic and the detailed collection of electronic medical records. The study also distinguishes itself as one of the first studies to assess the use of LVD in patients with POAG. The detailed demographics as well as analysis based on different categories of VI, visual field defect degree and severity of visual field defect, and the amount of visual improvement among glaucoma patients is also the highlight of the study. Optometrists in low-vision clinics make sure that patients are advised to either purchase devices or glasses. Enough training was given to the patient and the attendant to operate/use devices. These patients are also encouraged to contact the department in case of any user doubts. Other studies have looked at the preference pattern of LVD in all types of glaucoma. There is a parity of literature regarding the degree of improvement with LVD.

However, the fact that the study is retrospective presents one of the drawbacks. Another limiting factor happens to be the fact that the study does not contain any longitudinal data, which would be much more helpful in understanding the role of LVD concerning the progression of POAG.

Despite glaucoma being the second leading cause of blindness, the number of patients with glaucoma attending low-vision services is low [23]. The American Academy of Ophthalmology, in their preferred practice patterns, has guidelines for referral of patients with best corrected visual acuity <20/40, visual field defect, and decreased contrast sensitivity to low-vision services [24]. Pabon et al. reported that glaucoma patients constituted only 13.9% of those enrolled in low-vision services [17]. They postulated that this could be due to a low referral due to inadequate knowledge regarding the LVD services. Patients are often referred when the physicians feel that nothing further can be done [25]. Loss of visual field was the second most common clinician-based referral criteria in the study by Kaleem et al. [23]. We hope that our results will encourage practitioners to refer patients with POAG to the low-vision clinic early in the course of the disease.

## Conclusions

This study provides a detailed exploration of the clinical profiles of patients with POAG and the efficacy of LVD in enhancing visual outcomes. The findings reveal that a significant proportion of patients (82.5%) suffered from severe to total blindness, with notable improvements in near vision achieved through LVD, particularly among those with advanced visual field defects. Additionally, the research highlights the impact of socioeconomic status, with financially independent patients showing greater benefits from LVD interventions. This research is invaluable as it emphasizes the critical need for early referral to low-vision services and underscores the transformative potential of LVD in the visual rehabilitation of POAG patients. By bringing these insights to light, this study not only contributes to the field but also aims to inspire ongoing efforts to improve the quality of life and functional capabilities of those living with this condition.
